# Does the Fractionalization of Daily Physical Activity (Sporadic vs. Bouts) Impact Cardiometabolic Risk Factors in Children and Youth?

**DOI:** 10.1371/journal.pone.0025733

**Published:** 2011-10-05

**Authors:** Rebecca M. Holman, Valerie Carson, Ian Janssen

**Affiliations:** 1 School of Kinesiology and Health Studies, Queen's University, Kingston, Ontario, Canada; 2 Department of Community Health and Epidemiology, Queen's University, Kingston, Ontario, Canada; University of Granada, Spain

## Abstract

**Objective:**

Children and youth accumulate their daily moderate-to-vigorous physical activity (MVPA) in bouts (i.e., ≥5 consecutive minutes) and in a sporadic manner (i.e., <5 consecutive minutes). The study objective was to determine, within children and youth, whether MVPA accumulated in bouts is more strongly associated with cardiometabolic risk factors than an equivalent volume of MVPA accumulated sporadically.

**Methods:**

Participants consisted of 2754 children and youth aged 6–19 years from the 2003–2006 National Health and Nutrition Examination Survey, a representative cross-sectional study. Bouts and sporadic MVPA were measured objectively over 7 days using Actigraph accelerometers. Thresholds of 5 and 10 consecutive minutes were used to differentiate between bouts and sporadic MVPA. A high cardiometabolic risk factor score (CRS) was created based on measures of waist circumference, non-high-density lipoprotein cholesterol, C-reactive protein, and systolic blood pressure. Associations were examined using logistic regression and controlled for covariates (sex, age, ethnicity, socioeconomic status, dietary fat and sodium, smoking, and accelerometry wear time).

**Results:**

The odds of a high CRS decreased in a dose-response for both the sporadic and bout MVPA measures. Relative to quartile 1, the odds ratio (95% confidence interval) for a high CRS in quartile 4 was 0.25 (0.10–0.60) for sporadic MVPA, 0.17 (0.09–0.34) for ≥5 minute bouts of MVPA, and 0.19 (0.11–0.34) for ≥10 minute bouts of MVPA. The sporadic and bout MVPA measures had a similar ability to distinguish between participants with high and normal CRS. Relative to 0 minutes of MVPA, an equivalent number of minutes of sporadic MVPA and bouts of MVPA had an almost identical odds ratio for a high CRS. The findings were consistent for 5 and 10 minute bout thresholds.

**Conclusions:**

The relations between sporadic MVPA and bouts of MVPA with cardiometabolic risk factors were remarkably similar in children and youth.

## Introduction

The cardiovascular, metabolic, and overall health benefits that children and youth achieve by engaging in moderate-to-vigorous intensity physical activity (MVPA) are well appreciated [1]. Within the past 3 years, new evidence-based physical activity guidelines for school-aged children and youth have been released by the World Health Organization [2] and health authorities in the U.S. [3] and Canada [4]. These guidelines provide public health targets for the appropriate type, amount, intensity, and patterns of physical activity needed to achieve good health. The primary recommendation within all three sets of guidelines is that children and youth should accumulate 60 or more minutes of MVPA on a daily basis.

The 60 minute per day dose of MVPA that has been recommended for children and youth is different than the 150 minutes per week dose of MVPA that is the foundation of the adult physical activity guidelines [2], [3], [4]. Another key difference between the child/youth and adult guidelines, which is the focus of the present study, is the pattern in which the recommended dose of MVPA needs to be accumulated. Within the adult guidelines there is a stipulation that the MVPA needs to be accumulated in bouts lasting at least 10 minutes. There is no such stipulation in the child and youth guidelines. This reflects a lack of studies that have investigated the relevance of bouts of physical activity in the pediatric age group [1].

It is important to study the influence of MVPA patterns on the health of children and youth because sporadic activity, defined here as activity accrued in less than 5 consecutive minutes, is more characteristic of children and youth than adults [5]. In fact, recent research indicates that 66% of the total MVPA in 8–17 year olds is accumulated in a sporadic manner [6]. The purpose of this study was to determine whether bouts of MVPA influence health, in this case cardiometabolic risk factors, to a greater extent than an equivalent volume of sporadic MVPA.

Cardiometabolic risk factors refer to traditional and non-traditional risk factors for cardiovascular disease and type 2 diabetes [7]. Modifiable cardiometabolic risk factors include measures of abdominal obesity, lipid/lipoprotein factors, high blood pressure, and inflammatory markers [7]. The primary outcome for this study was a summary score based on four cardiometabolic risk factors. This summary score captured different types of cardiometabolic risk factors and was based upon waist circumference, non-high-density lipoprotein cholesterol, systolic blood pressure, and C-reactive protein measures.

## Methods

### Ethics Statement

Consent was obtained from all participants and their parents/guardian if <18 years old. The National Health and Nutrition Examination Survey (NHANES) was approved by the National Center for Health Statistics. The analyses presented here were approved by the Queen's University Health Sciences Research Ethics board.

### Participants

This study was based on the 2003/04 and 2005/06 NHANES cycles. This is a cross-sectional survey that included interviews and physical exams conduced in a mobile exam center. Participants between 6 and 19 years old who had complete measures of interest were eligible for this study, resulting in 2754 participants (1399 males, 1355 females, 954 children aged 6–11 years, 1800 adolescents aged 12–19 years).

### Physical Activity

MVPA was measured using Actigraph AM-7124 accelerometers. This device is a uni-axial accelerometer and recorded average acceleration and intensity of activity over one-minute intervals. Participants were asked to wear the accelerometer on their right hip for 7 consecutive days following their mobile exam center visit, except when sleeping or when the accelerometer could get wet. Accelerometers were mailed back to the study investigators in pre-paid envelopes. Data from the accelerometers was downloaded by survey collaborators and checked for outliers and unreasonable values, which were removed.

Additional data reduction was completed by the authors. Initially, we removed days with incomplete information. A day was considered complete if it contained ≥10 hours of wear time [8], [9], [10], non-wear time was defined as a period of >20 minutes of zero counts [8], [9], [10]. The second data reduction step involved removing participants with an insufficient number of days of complete data. Based on existing precedence for using accelerometry data, only participants with ≥4 complete days, including one weekend day (because children exhibit different activity levels on the weekends compared to weekdays [11]), were included [8], [9], [10]. The test-retest reliability of 4 days of accelerometer measurement in children and youth is 0.7 [12].

After the accelerometry data reduction was complete, we estimated the average daily minutes of sporadic MVPA, bouts of MVPA, and total (sporadic + bouts) MVPA. All of the MVPA variables were calculated for each day with acceptable accelerometry data (i.e., >10 hours of wear time), and then averaged over the number of days with acceptable data. The initial part of this process involved determining which of the one minute epochs corresponded to MVPA. A regression equation developed by Freedson and colleagues for 6–18 year olds was used to estimate metabolic equivalents (METs) for each epoch count per minute value based on the epoch count per minute value and the participants age [13]. Epoch values that were equivalent to ≥4.0 METs were considered to be minutes of MVPA [14]. Because there is no accepted precedence on the minimal or optimal bout length, we defined bouts and sporadic MVPA using two different thresholds for the minimal bout length: ≥5 minutes and ≥10 minutes. We also considered a 15 minute threshold; however, preliminary analyses revealed that 40% of the study sample did not have any ≥15 minute MVPA bouts over the 7 day measurement period.

Based on the 5 minute bout threshold, sporadic MVPA was calculated as that sum of all MVPA that was performed in less than 5 consecutive minutes (i.e., <5 consecutive epoch values above the MVPA threshold). Bouts of MVPA were calculated as the sum of all MVPA that was performed in 5 or more consecutive minutes (i.e., ≥5 consecutive epoch values above the MVPA threshold). The bout stopped being recorded when 80% of it (e.g., 8 out of 10 minutes) was no longer above the MVPA threshold MVPA. The 80% threshold accounted for the temporary rest periods during MVPA activities. The bout also stopped if there were 5 consecutive minutes below the MVPA threshold.

Based on the 10 minute bout threshold, sporadic MVPA was calculated as that sum of all MVPA that was performed in less than 10 consecutive minutes. Bouts of MVPA were calculated as the sum of all MVPA that was performed in 10 or more consecutive minutes. The bout stopped being recorded when 80% of it was no longer above the MVPA threshold or if there were 5 consecutive minutes below the MVPA threshold.

### Cardiometabolic Risk Factors

Waist circumference, systolic blood pressure, non-high-density lipoprotein cholesterol (non-HDL cholesterol), and C-reactive protein were the cardiometabolic risk factors studied. They were selected based on their availability in the NHANES dataset and because they capture different aspects of cardiometabolic risk. All measurements were taken by trained personnel at the mobile examination center visit. Triglycerides and fasting glucose were not examined as cardiometabolic risk factors due to the unavailability of fasting values in participants under the age of 12 in the NHANES dataset.

Waist circumference was measured to the nearest 0.1 cm at the level of the iliac crest. Waist circumference is an effective measure of abdominal adiposity among children and adolescents and is a better predictor of cardiometabolic risk factors than the body mass index [15]. Blood pressure was measured manually four consecutive times on the right arm while seated. We calculated the average blood pressures. We calculated non-HDL cholesterol by subtracting HDL cholesterol from total cholesterol [16]. HDL cholesterol was measured using the direct HDL immunoassay method and total cholesterol was measured enzymatically in serum in a series of coupled reactions using cholesteryl ester hydrolase, cholesterol oxidase, and peroxidase. Non-HDL cholesterol was chosen as the lipid marker because it is an important indicator of cardiovascular disease and diabetes risk among children and adolescents that it is not reliant upon a fasting blood sample [17]. C-reactive protein was measured by latex enhanced nephelometry. C-reactive protein was chosen as the inflammatory marker because of its availability in the dataset, known impact on cardiovascular disease, and because it is not reliant upon a fasting blood sample [18], [19].

Waist circumference and non-HDL cholesterol were not normally distributed so they were log transformed by the authors prior to analyses. Age-adjusted values were created by the authors for each of the cardio-metabolic risk factors because they change with normal growth and maturation [20]. Using forward stepwise regression, each of the cardio-metabolic risk factors were regressed up to a full cubic polynomial in age (age, age^2^, age^3^) separately within males and females. Variables were allowed to enter or leave the model at *P*<0.10. The standardized residuals were retained, and used to represent the age-adjusted values. Participants were then ranked based on the residual for each risk factor. A mean of the ranks was used to represent a summary cardiometabolic risk score (CRS). Blood pressure was not measured in children <8 years old, so the CRS for 6–7 year olds was limited to three risk factors. We categorized CRS into quartiles; the highest quartile denotes a high CRS.

### Covariates

Age, gender, ethnicity (non-Hispanic white, non-Hispanic black, Hispanic, other), socioeconomic status, smoking status, and diet were considered as covariates. The poverty-to-income ratio, provided within the NHANES dataset, is a ratio between family income and poverty threshold and was used to measure socioeconomic status [21]. Smoking was assessed in NHANES by asking participants 12 and older, “Have you ever tried cigarette smoking, even 1 or 2 puffs?” We grouped participants into “yes” or “no” categories. Participants <12 years old were placed into the “no” category. Diet intake was obtained via a 24 hour food recall obtained through an in-person interview conducted by a dietician. Dietary food recall data were imported into a computer-assisted food coding and data management system to determine total nutrient intakes (Agricultural Research Service, USDA Food and Nutrient Database for Dietary Studies 2.0). From this, we created two binary variables: total fat (≤35% or >35% total calories) and sodium (≤2300 or >2300 mg/day) [22].

### Statistical Analysis

Analyses were completed using SAS version 9.2 (SAS Inc., Carry, N.C.), which accounted for NHANES sample design. All analyses were initially performed based on the 5 minute bout threshold and were repeated based on the 10 minute bout threshold. Conventional descriptive statistics were used to describe the study variables. Partial correlations (adjusted for sex, ethnicity, and age) were used to examine relations between continuous MVPA measures. Logistic regression models were used to examine the association between the MVPA measures with a high summary CRS (i.e., scores in the top quartile). All logistic regression models were adjusted for sex, age, ethnicity, socioeconomic status, diet (total fat and sodium), smoking status, and total accelerometry wear time.

In an initial set of logistic regression models, each of the three MVPA (total, sporadic, bouts) variables were included as categorical variables. The MVPA categories were based on quartiles. To compare the discriminatory ability of these models (e.g., the ability to separate those participants with a high CRS from those with normal values), their *c* statistic values were determined [23]. The *c* statistic is identical to the area under the receiver operating characteristic (ROC) curve, with values ranging from 0.5 (no better discrimination than chance alone) to 1.0 (perfect discrimination). A second set of logistic regression models was based on continuous measures of the sporadic MVPA and total bouts of MVPA variables. Linear (min/day), quadratic (min/day, min/day^2^), and cubic (min/day, min/day^2^, min/day^3^) polynomial models were tested for each of these continuous MVPA variables.

## Results

Descriptive characteristics of the 2754 participants are shown in Table 1. A breakdown of these characteristics by gender and age is shown in the Table S1. The median age of the sample was 13 years and 50.8% were male. The median total MVPA in the sample was 33 minutes per day. The median amount of MVPA accrued in bouts was 15 minutes per day when based on the 5 minute threshold and 8 minutes per day when based on the 10 minute threshold. Of the total sample, 87.5% participated in at least one 5 minute bout and 72.5% participated in at least one 10 minute bout over the accelerometer measurement period. Approximately 11% of the sample had 4 valid days of accelerometer wear time, 20% had 5 valid days, 35% had 6 valid days, and 34% had 7 valid days.

**Table 1:** Participant characteristics (N = 2754).

Variable	Total(N = 2754)
Age, years	13 (10–16)
Sex (%)	
Male	50.8
Female	49.2
Race (%)	
Hispanic	37.7
Non-Hispanic white	24.2
Non-Hispanic black	33.4
Other	4.7
Current smoker (%)	22.7
High fat diet (%)	40.8
High sodium diet (%)	71.5
Cardiometabolic risk factors	
Waist circumference, cm	73.1 (64.6–83.3)
Non-HDL cholesterol, mmol/l	2.7 (2.3–3.2)
C-reactive protein, mmol/l	0.002 (0.001–0.007)
Systolic blood pressure, mmHg	107 (101–114)
Moderate-to-vigorous physical activity	
*Bouts define using 5* *min threshold*	
Total (sporadic + bouts), min/day	33 (14–70)
Sporadic (1–4 min), min/day	20 (9–39)
Bouts (≥5 min), min/day	15 (4–38)
*Bouts defined using 10* *min threshold*	
Total (sporadic + bouts), min/day	33 (14–70)
Sporadic (1–9 min), min/day	26 (11–50)
Bouts (≥10 min), min/day	8 (0–24)

Data presented as median (inter-quartile range) for continuous variables or prevalence (%) for categorical variables.

Partial correlations (adjusted for sex, ethnicity, and age) between the MVPA measures are shown in Table 2. Irrespective of what threshold was used to define a bout of MVPA, total MVPA was highly correlated (r  = 0.80 to 0.92) with the sporadic and bout MVPA measures. The correlations between the sporadic and bout MVPA measures were not as strong (r = 0.43 to 0.51).

**Table 2:** Partial correlations (adjusted for sex, ethnicity, and age) among moderate-to-vigorous physical activity measures (N = 2754).

	Sporadic	Bouts
*Bouts defined using 5 minute threshold*		
Total	0.80	0.92
Sporadic	--	0.51
*Bouts defined using 10 minute threshold*		
Total	0.86	0.83
Sporadic	--	0.43

Irrespective of what threshold was used to define a bout of MVPA, the relative odds of a high CRS decreased in a dose-response manner (*P_trend_*≤0.02) when moving from quartile 1 to quartile 4 for the total, sporadic, and bout MVPA measures (Table 3). For example, relative to quartile 1, the odds ratio (95% confidence interval) for a high CRS was 0.25 (0.10–0.60) in quartile 4 for the sporadic MVPA measure, 0.17 (0.09–0.34) for quartile 4 for the bout MVPA measure when based upon a 5 minute bout threshold, and 0.19 (0.11–0.34) for quartile 4 for the bout MVPA measure when based upon a 10 minute bout threshold. A comparison of the C-statistic values suggests that each of the total, sporadic, and bout MVPA measures had a similar ability to distinguish between participants with a high and normal CRS.

**Table 3:** Relations between moderate-to-vigorous physical activity measures and elevated levels of the summary cardiometabolic risk factor score (N = 2754).

Moderate-to-vigorousphysical activity measure	Prevalencewith high score, %	Adjusted odds ratio(95% confidence interval)*	C-statistic
*Total (sporadic + bouts)*			0.61
Quartile 1 (0–13 min/day)	32.3	1.00	
Quartile 2 (14–33 min/day)	24.5	0.56 (0.35–0.90)	
Quartile 3 (34–70 min/day)	24.8	0.37 (0.21–0.66)	
Quartile 4 (71–249 min/day)	18.6	0.19 (0.08–0.46)	
	P_trend_<0.01	P_trend_<0.01	
*Bouts defined using 5 minute threshold*			
Sporadic (1–4 minutes)			0.60
Quartile 1 (0–9 min/day)	30.7	1.00	
Quartile 2 (10–20 min/day)	25.2	0.70 (0.43–1.13)	
Quartile 3 (21–39 min/day)	25.1	0.56 (0.32–0.98)	
Quartile 4 (40–119 min/day)	19.2	0.25 (0.10–0.60)	
	P_trend_<0.01	P_trend_ = 0.02	
Bouts (≥5 consecutive minutes)			0.62
Quartile 1 (0–4 min/day)	32.4	1.00	
Quartile 2 (5–15 min/day)	26.5	0.51 (0.33–0.77)	
Quartile 3 (16–38 min/day)	23.1	0.37 (0.22–0.63)	
Quartile 4 (39–218 min/day)	18.3	0.17 (0.09–0.34)	
	P_trend_<0.01	P_trend_<0.01	
*Bouts defined using 10 minute threshold*			
Sporadic (1–9 minutes)			0.61
Quartile 1 (0–10 min/day)	31.1	1.00	
Quartile 2 (11–25 min/day)	25.2	0.65 (0.40–1.04)	
Quartile 3 (26–49 min/day)	25.0	0.53 (0.30–0.93)	
Quartile 4 (50–183 min/day)	19.0	0.25 (0.11–0.60)	
	P_trend_<0.01	P_trend_ = 0.02	
Bouts (≥10 consecutive minutes)			0.62
Quartile 1 (0–min/day)	32.0	1.00	
Quartile 2 (1–8 min/day)	26.2	0.64 (0.42–0.99)	
Quartile 3 (9–24 min/day)	23.5	0.45 (0.28–0.70)	
Quartile 4 (25–186 min/day)	17.6	0.19 (0.11–0.34)	
	P_trend_<0.01	P_trend_<0.01	

*Adjusted for sex, age, ethnicity, socioeconomic status, diet, smoking, and total accelerometer wear time.

Age (6–11 year old children vs. 12–19 year old adolescents) and gender interactions were tested for the analyses shown in Table 3. There was only one meaningful interaction; this was an age interaction for total (sporadic + bouts) MVPA wherein the associations were stronger in children than in adolescents. Specifically, for 6–11 year olds the odds ratios and 95% confidence intervals were 1.00 in quartile 1, 0.18 (0.02–2.05) in quartile 2, 0.08 (0.01–0.96) in quartile 3, and 0.04 (0.01–0.50) in quartile 4. The corresponding values for 12.19 year olds were 1.00, 0.51 (0.34–0.88), 0.36 (0.18–0.71), and 0.40 (0.14–1.12).

Similar relationships to those presented in Table 3 for a high CRS were observed for the 3 of the individual cardiometabolic risk factors (see results for non-HDL cholesterol, CRP, and systolic blood pressure in Table 4). However, the odds ratio gradient for a high waist circumference was noticeably stronger across MVPA bout quartiles than sporadic MVPA quartiles.

**Table 4:** Relations between moderate-to-vigorous physical activity measures and elevated levels of the individual cardiometabolic risk factors (N = 2754).

Moderate-to-vigorousphysical activity measure			WC,OR (95% CI)*	Non-HDL,OR (95% CI)*	CRP,OR (95% CI)*	SBP,OR (95% CI)*
*Total (sporadic + bouts)*						
Quartile 1	1.00			1.00	1.00	1.00
Quartile 2	0.65 (0.42–1.03)			0.58 (0.36–0.95)	0.89 (0.56–1.43)	0.50 (0.30–0.83)
Quartile 3	0.44 (0.24–0.80)			0.72 (0.42–1.25)	0.44 (0.24–0.81)	0.51 (0.29–0.92)
Quartile 4	0.21 (0.09–0.51)			0.42 (0.20–0.90)	0.36 (0.16–0.79)	0.52 (0.22–1.19)
*Bouts defined using 5 minute threshold*						
Sporadic (1–4 minutes)						
Quartile 1		1.00		1.00	1.00	1.00
Quartile 2		1.09 (0.69–1.75)		0.72 (0.44–1.18)	0.96 (0.61–1.50)	0.61 (0.36–1.00)
Quartile 3		0.75 (0.41–1.35)		0.76 (0.43–1.32)	0.60 (0.35–1.04)	0.41 (0.24–0.72)
Quartile 4		0.47 (0.20–1.10)		0.52 (0.24–1.14)	0.33 (0.16–0.68)	0.37 (0.16–0.85)
Bouts (≥5 consecutive minutes)						
Quartile 1	1.00			1.00	1.00	1.00
Quartile 2	0.62 (0.40–0.96)			0.54 (0.34–0.86)	0.76 (0.49–1.19)	0.47 (0.30–0.75)
Quartile 3	0.41 (0.24–0.70)			0.86 (0.52–1.42)	0.39 (0.23–0.67	0.70 (0.41–1.17)
Quartile 4	0.22 (0.11–0.44)			0.49 (0.26–0.93)	0.42 (0.21–0.85)	0.53 (0.27–1.02)
*Bouts defined using 10 minute threshold*						
Sporadic (1–9 minutes)						
Quartile 1	1.00			1.00	1.00	1.00
Quartile 2	0.87 (0.54–1.39)			0.64 (0.39–1.05)	0.83 (0.52–1.33)	0.72 (0.42–1.21)
Quartile 3	0.66 (0.37–1.20)			0.78 (0.44–1.37)	0.43 (0.23–0.79)	0.48 (0.28–0.82)
Quartile 4	0.42 (0.18–0.95)			0.55 (0.25–1.19)	0.31 (0.14–0.69)	0.47 (0.21–1.07)
Bouts (≥10 consecutive minutes)						
Quartile 1	1.00			1.00	1.00	1.00
Quartile 2	0.76 (0.49–1.17)			0.73 (0.47–1.15)	0.75 (0.48–1.18)	0.57 (0.36–0.91)
Quartile 3	0.51 (0.32–0.80)			0.82 (0.51–1.31)	0.65 (0.40–1.05)	0.84 (0.53–1.32)
Quartile 4	0.23 (0.13–0.43)			0.56 (0.31–1.00)	0.49 (0.27–0.91)	0.71(0.37–1.34)

WC  =  waist circumference, Non-HDL  =  Non-High Density Lipoprotein cholesterol, CRP  =  C-reactive protein, SBP  =  systolic blood pressure, OR (95% CI)  =  odds ratio (95% confidence interval).

*Adjusted for sex, age, ethnicity, socioeconomic status, diet, smoking, and total accelerometer weartime.

**Figure 1:**
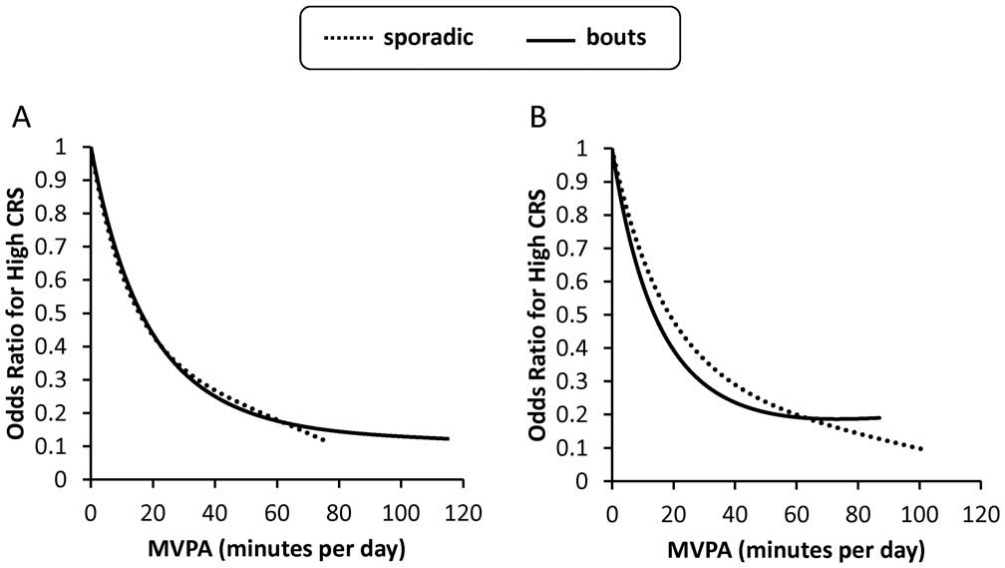
Association between sporadic and bouts of physical activity with high summary cardiometabolic risk factor scores . Odds ratio for a high cardiometabolic risk score (CRS) according to minutes per day of sporadic (dashed lines) and bouts (solid lines) of moderate-to-vigorous physical activity. In panel A (left), bouts were defined using a 5 minute threshold. In panel B (right), bouts were defined using a 10 minute threshold. Odds ratios were plotted from 0 minutes per day (referent) to values that corresponded to the 98^th^ percentile of the sample. Odds ratios were adjusted for sex, age, ethnicity, socioeconomic status, diet, smoking, and total accelerometer wear time.

As illustrated in Figure 1, an equivalent number of minutes of sporadic MVPA and bouts of MVPA had a comparable influence on the relative odds of a high CRS. This was true irrespective of whether the 5 minute (Panel A on left) and 10 minute (panel B on right) bout threshold was used. For instance, when the 5 minute bout threshold was used, the relative odds of a high CRS at 30 minutes per day relative to 0 minutes per day was 0.33 for sporadic MVPA and 0.32 for bouts of MVPA. Note that the associations in the figure were adjusted for the covariates. The results for sporadic MVPA were also adjusted for bouts of MVPA, and the results for bouts of MVPA were also adjusted for sporadic MVPA.

## Discussion

The primary objective of this study was to determine whether bouts of MVPA influence cardiometabolic risk factors to a greater extent than an equivalent volume of sporadic MVPA within children and youth. The key findings are: 1) the volume of MVPA accumulated in a sporadic manner was only moderately correlated (r ∼0.50) with the volume of MVPA accumulated in bouts, 2) the volume of both sporadic MVPA and bouts of MVPA were strongly associated with a high CRS, and 3) an equivalent volume of sporadic MVPA and bouts of MVPA had an almost identical effect on a high CRS, and this was true regardless of whether bouts were defined using a 5 or 10 minute threshold.

Previous accelerometry-based studies have reported that sporadic MVPA [6], [24] and bouts of MVPA [6], [25] are related to cardiometabolic risk factors within pediatric and adult populations. To our knowledge, only one study has attempted to determine if the associations with cardiometabolic risk factors are different for sporadic MVPA and bouts of MVPA. In that study, which was based on 8–17 year olds from the 2003/04 NHANES cycle (who accounted for ∼50% of the NHANES sample examined in here), the amount of MVPA spent in bouts lasting at least 5 minutes was a significant predictor of overweight after controlling for the total volume of MVPA [6]. This suggests that bouts of MVPA was a stronger overall predictor of overweight than sporadic MVPA in that study. Although this finding appears to contradict those of the present study, inspection of Tables 3 and 4 indicates otherwise. While MVPA accumulated in bouts was not a stronger predictor of a high summary CRS than MVPA accumulated in a sporadic manner (Table 3 and Figure 1) and the non-obesity CRS components (Table 4), this was not the case for the waist circumference (Table 4). For example, based on the 5 minute bout threshold, the odds ratio in quartile 4 for a high waist circumference was about twofold higher for sporadic MVPA than for bouts of MVPA (0.47 vs. 0.22). Collectively, these findings suggest that bouts of MVPA may have a greater impact on overweight and obesity than an equivalent volume of sporadic MVPA. This does not; however, appear to be the case for non-obesity cardiometabolic risk factors such as C-reactive protein, blood pressure, and non-HDL. At this time it is unclear as to why bouts of MVPA may have a particularly strong impact on overweight and obesity.

These results of our study have important implications for public health. The recently released World Health Organization [2], US [3], and Canadian [4] physical activity guidelines for children and youth recommend 60 minutes per day of MVPA. Unlike the adult physical activity guidelines, the child and youth guidelines do not include a stipulation that the MVPA needs to be accrued in bouts of at least 10 minutes in duration. The results of our study provide support for the dose and patterns components of the child and youth guidelines. Regarding the dose component, we observed a clear dose-response gradient between the total volume of MVPA and cardiometabolic risk such that the most active total MVPA quartile (>70 min/day of MVPA) was the least likely to have elevated cardiometabolic risk factor values. Regarding the pattern component, our findings suggest that equivalent volumes of sporadic and bouted MVPA have a similar impact on overall cardiometabolic risk.

This study has several strengths, including the large sample, the use of accelerometers to provide a valid and unbiased measure of MVPA patterns [26], [27], [28], and the inclusion of multiple cardiometabolic risk factors. However, as with any study, this study was not void of limitations. A major limitation was the cross-sectional nature of the study, which precludes us from making strong causal statements about the relations that were studied. Nonetheless, we are confident that the MVPA variables proceeded the CRS outcome in our study because previous prospective cohort and randomized controlled trails have reported similar relationships [1], [29],_ENREF_29 and because the associations observed in our study were strong, followed clear dose-response patterns, and were biologically plausible. Another important limitation is the fact that the uniaxial accelerometers used in NHANES did not capture water based activities (such as swimming) and were limited in their ability to capture some land-based activities (such as cycling and weight training) [30]. Assuming that the measurement error of the accelerometers was non-differential, the associations that we observed between physical activity and cardiometabolic risk would have been underestimated. Finally, a large percentage (58%) of the NHANES sample was excluded from the analyses because of incomplete or missing data. Although the exclusion of these participants influenced the descriptive information (Table 1), it is unlikely that this would have significantly influenced the associations between the MVPA and CRS variables (Tables 3 and 4, Figure 1).

In conclusion, the findings of this study confirm that MVPA is related to cardiometabolic risk factors within children and youth, and suggest that these relationships are comparable for equivalent doses of sporadic MVPA and bouts of MVPA. Tightly regulated randomized control trials are needed to confirm the results of this study.

## Supporting Information

Table S1 **Participant characteristics by gender and age.** Data presented as median (inter-quartile range) for continuous variables or prevalence (%) for categorical variables.
(DOCX)
