# Supplementary material for: Does the Fractionalization of Daily Physical Activity (Sporadic vs. Bouts) Impact Cardiometabolic Risk Factors in Children and Youth?
Source: PLoS One. 2011 Oct 5;6(10):e25733. doi: 10.1371/journal.pone.0025733 (PMC3187782; doi:10.1371/journal.pone.0025733)
Supplement: Table S1 — Participant characteristics by gender and age. Data presented as median (inter-quartile range) for continuous variables or prevalence (%) for categorical variables. (DOCX) [file pone.0025733.s001.docx]

**Table S1.** Participant characteristics by gender and age.

| Variable | Males  (N=1399) | Females  (N=1355) | 6-11 year olds  (N=954) | 12-19 year olds  (N=1800) |
| --- | --- | --- | --- | --- |
| Age, years | 13 (10-16) | 13 (10-16) | 9 (7-10) | 15 (13-17) |
| Sex (%) |  |  |  |  |
| Male | 100 | 0 | 49.3 | 51.6 |
| Female | -- | -- | 50.7 | 48.4 |
| Race (%) |  |  |  |  |
| Hispanic | 36.8 | 38.7 | 39.7 | 36.7 |
| Non-Hispanic white | 23.2 | 25.2 | 25.0 | 23.7 |
| Non-Hispanic black | 35.6 | 31.2 | 28.8 | 35.9 |
| Other | 4.4 | 4.9 | 6.5 | 3.7 |
| Current smoker (%) | 23.8 | 21.6 | 0 | 34.8 |
| High fat diet (%) | 40.2 | 41.3 | 41.1 | 40.1 |
| High sodium diet (%) | 78.2 | 64.6 | 69.5 | 72.6 |
| Cardiometabolic risk factors |  |  |  |  |
| Waist circumference, cm | 72.2 (63.4-83.3) | 73.8 (65.5-83.2) | 61.9 (56.2-71.0) | 76.9 (70.6-86.9) |
| Non-HDL cholesterol, mmol/l | 2.7 (2.3-3.2) | 2.7 (2.3-3.3) | 2.8 (2.3-3.3) | 2.7 (2.2-3.2) |
| C-reactive protein, mmol/l | 0.002 (0.001-0.007) | 0.003 (0.001-0.009) | 0.002 (0.001-0.007) | 0.003 (0.001-0.008) |
| Systolic blood pressure, mmHg | 109 (102-116) | 105 (99-112) | 101 (96-107) | 109 (102-116) |
| Moderate-to-vigorous physical activity |  |  |  |  |
| *Bouts define using 5 min threshold* |  |  |  |  |
| Total (sporadic + bouts), min/day | 45 (21-83) | 25 (8-55) | 83 (54-115) | 20 (8-36) |
| Sporadic (1-4 min), min/day | 24 (12-42) | 16 (6-34) | 47 (33-60) | 12 (5-21) |
| Bouts (≥5 min), min/day | 23 (8-50) | 9 (2-26) | 40 (22-66) | 8 (1-20) |
| *Bouts defined using 10 min threshold* |  |  |  |  |
| Total (sporadic + bouts), min/day | 45 (21-83) | 25 (8-55) | 83 (54-115) | 20 (8.36) |
| Sporadic (1-9 min), min/day | 31 (16-56) | 20 (7-42) | 60 (42-78) | 15 (7-27) |
| Bouts (≥10 min), min/day | 13 (3-33) | 4 (0-14) | 24 (11-44) | 3 (0-12) |

Data presented as median (inter-quartile range) for continuous variables or prevalence (%) for categorical variables.
